# Incomplete basic vaccination and associated factors among children aged 12–23 months in resource-limited countries: a spatial and multilevel regression analysis of recent DHS data from 48 countries

**DOI:** 10.3389/fpubh.2025.1463303

**Published:** 2025-04-14

**Authors:** Mihret Getnet, Melak Jejaw, Tadele Biresaw Belachew, Banchlay Addis, Endalkachew Dellie, Tesfahun Zemene Tafere, Nigusu Worku, Demiss Mulatu Geberu, Lake Yazachew, Getachew Teshale, Misganaw Guadie Tiruneh, Kaleb Assegid Demissie

**Affiliations:** ^1^Department of Human Physiology, School of Medicine, College of Medicine and Health Sciences, University of Gondar, Gondar, Ethiopia; ^2^Department of Epidemiology and Biostatistics, Institute of Public Health, College of Medicine and Health Sciences, University of Gondar, Gondar, Ethiopia; ^3^Department of Health Systems and Policy, Institute of Public Health, University of Gondar, Gondar, Ethiopia

**Keywords:** incomplete, vaccination, children, resource limited, countries

## Abstract

**Background:**

Childhood basic vaccinations are a cost-effective and essential preventive health strategy globally in resource-limited nations. The United Nations Sustainable Development Goals aim to reach these ambitious targets, making it crucial to identify underserved populations and address the barriers they face in accessing life-saving immunizations. To date, no spatial analyses have been performed to identify areas of hotspots of incomplete basic vaccination among children in resource-limited countries globally. Therefore, determining the geographic distribution of incomplete basic vaccinations and associated factors is important for prioritizing intervention programs in resource-limited countries.

**Objective:**

This study aims to assess incomplete basic vaccinations and associated factors among children aged 12–23 months in resource-limited countries based on the recent Demographic and Health Survey (DHS) data of 48 countries.

**Methods:**

Data for the study were drawn from the DHS, a nationally representative cross-sectional survey conducted by considering the era of Millennium Development Goals and Sustainable Development Goals. A total of 48 resource-limited countries and a total weighted sample of 202,029 children (12–23 months) were included in our study. The data extraction, recoding, and analysis were conducted using STATA V.17. For the spatial analysis (spatial distribution, autocorrelation, and hotspot), ArcGIS version 10.7 software was used, and for the SaTScan analysis, SaTScan version 10.1 software was used. Descriptive statistics were presented using frequency tables and percentages. We employed multilevel logistic regression to investigate the associated factors of incomplete basic vaccination. In the multivariable analysis, variables with a *p*-value of ≤0.05 are considered significant factors associated with incomplete basic vaccination among children aged 12–23 months.

**Results:**

The overall incompleteness of basic vaccination among children in resource-limited countries was 51% (95%CI: 50–51%). The spatial analysis revealed that the incomplete basic vaccination among children significantly varied across resource-limited countries (Global Moran’s I = 0.208468, *p* < 0.001). The most likely clusters were located in Nigeria, Chad, Cameroon, and Niger, which were centered at (2.028929N, 15.135990 E)/1425.16 km radius, with a Log-Likelihood Ratio (LLR) of 3519.48 and a Relative Risk (RR) of 1.38 at *p*-value <0.001. Based on the final model of multilevel analysis, the following variables were statistically significant in relation to incomplete basic vaccination: age, marital status, maternal education, husband’s education, maternal occupation, media exposure, wealth index, antenatal care (ANC) visits, birth order, place of delivery, mode of delivery, health insurance coverage, perception of distance from a health facility, place of residence, community media exposure, community education, and country-level income status.

**Conclusion and recommendations:**

The spatial distribution of incomplete basic vaccination was significantly varied across the resource-limited countries. Both individual- and community-level factors were significantly associated with incomplete basic vaccination. Therefore, the World Health Organization and other stakeholders involved in child healthcare should work together to expand childhood vaccination and prioritize the hotspot areas of developing countries.

## Introduction

### Statement of the problem

Childhood basic vaccinations are a cost-effective and essential preventive health strategy on a global scale (1, 2). These vaccinations play a significant role in reducing the incidence and impact of common communicable diseases among children, ultimately lessening the economic and healthcare burdens on nations. Vaccinations targeting diseases such as tetanus, pertussis, diphtheria, polio, hepatitis B, and Hemophilus influenza type B have been used to avert millions of deaths annually and prevent disability in hundreds of thousands of children (3, 4). The United Nations Sustainable Development Goals (SDGs) prioritize equity and inclusivity, aiming to ensure that no one is left behind (2). However, an analysis of the SDG indicators and targets reveals a focus on national averages rather than specific attention to disadvantaged or marginalized populations.

According to a report by the World Health Organization (WHO), global immunization coverage has stagnated far below the 90% target for over a decade, meaning that a significant proportion of the global annual birth cohort remains incompletely vaccinated or unvaccinated (5). The global coverage of the four core vaccines: Bacillus Calmette-Guérin (BCG) vaccine, diphtheria-tetanus-pertussis vaccine (DTP), polio vaccine, and measles vaccine has increased from <5% to ≥84%. However, despite the availability of new vaccines for diseases such as rotavirus and pneumococcal diseases, vaccine-preventable illnesses still account for approximately 25% of the 10 million deaths that occur annually among children under 5 years old. This is partly due to the increasing number of infectious diseases that can currently be prevented through vaccination. Therefore, with improved vaccination coverage and the introduction of new vaccines, a larger proportion of children can be safeguarded against a wider array of infectious diseases. Despite these initiatives, the WHO noted in 2021 that 18.2 million children were not vaccinated, 5.9 million infants missed their first dose of the diphtheria, tetanus, pertussis (DTP) vaccine, and 6.8 million children received only partial vaccination (6).

In 2021, the Immunization Agenda of 2030 was introduced to enhance global access to vaccines and promote greater vaccine equity. The agenda aims to achieve a minimum of 90% coverage of essential childhood vaccines worldwide and reduce the number of completely unvaccinated children by 50% (7). To reach these ambitious targets, it is crucial to identify underserved populations and address the barriers they face in accessing life-saving immunizations (8). While national-level statistics on vaccine coverage are commonly reported, they may mask significant disparities at sub-national and socioeconomic levels (9). Identifying regions with high concentrations of under-vaccinated or unvaccinated children is essential to bridging the vaccination gap between economically disadvantaged and affluent nations.

Evidence from studies conducted previously attributes incomplete childhood vaccination, which identified different associated factors such as socioeconomic status disparities, residential areas, mother’s level of education, number of children in a family, birth weight, number of ANC visits, mother’s age, and media exposure (10–12). Limited evidence attributes incomplete vaccination among children to factors such as vaccine stock-outs, lack of awareness of the vaccination schedule by mothers or caregivers, and non-attendance of antenatal care during pregnancy (13).

Vaccines are effective in preventing illness, disability, and death caused by vaccine-preventable diseases such as cervical cancer, diphtheria, hepatitis B, measles, mumps, pertussis, pneumonia, polio, rotavirus diarrhea, rubella, and tetanus (14). Despite this, global vaccination coverage remains stable and currently prevents an estimated 2 to 3 million deaths annually. However, an estimated 21.8 million infants worldwide are still not receiving essential vaccines (7).

Adequate and equitable access to vaccines is important for attaining not only the global vaccine action plan targets but also the universal health coverage agenda and goal 3 of the Sustainable Development Goals (SDGs) by 2030 (11). Understanding the burden and associated factors of incomplete vaccination has policy and practice implications. It will inform contextually appropriate and locally responsive vaccination strategies and interventions that can redress persistent inequities in vaccination access. Therefore, the study contributes by presenting geographical variation and factors associated with incomplete basic vaccination among children aged 12–23 months in resource-limited countries globally.

## Methods and materials

### Study design

This is a cross-sectional analysis of globally representative data from the world’s recent Demographic and Health Survey (DHS) of each country. The main purpose of the DHS was to provide up-to-date estimates of demographic and health indicators, which include fertility levels, maternal and childhood mortality, immunization coverage, HIV testing and counseling, and physical and sexual violence against women.

### Sampling technique and data collection

**Figure 1:**
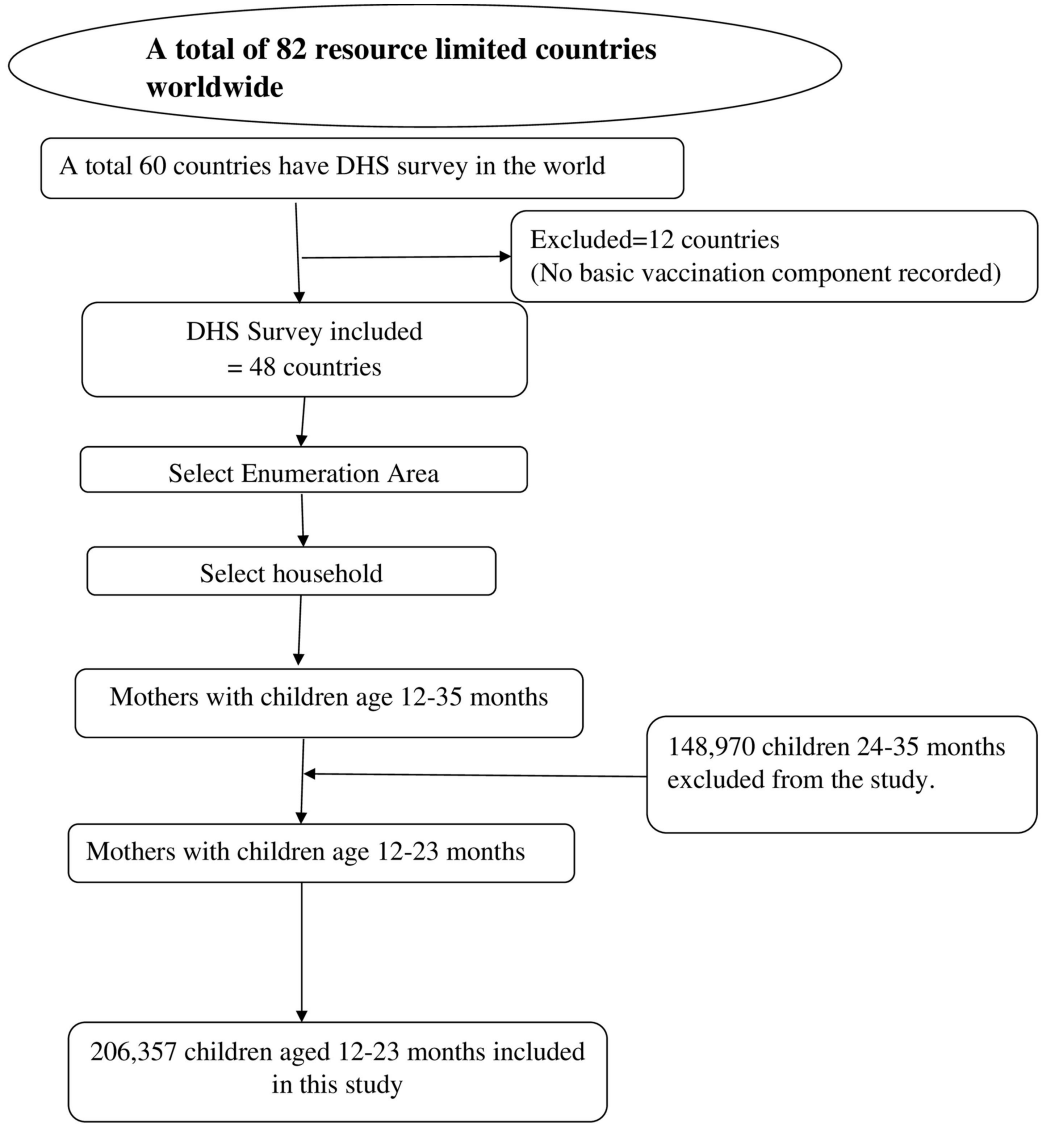
Flow diagram showing sampling procedure of DHS data.

The Demographic Health Survey (DHS) follows a multistage stratified random sampling technique. A two-stage stratified cluster sampling was used. In the first stage, the number of households needed per geographical area is determined, and clusters (or census enumeration areas) are randomly selected with a probability proportional to size. A random selection of the households in the chosen clusters was used for the second stage. Standard model questionnaires are then used to gather primary data at the household and individual levels for each sampled household. The list of all enumeration areas (EAs), which are geographic areas of 200–300 houses, was used to count units for the census. In the first stage, the EAs were selected with a probability proportional to the EA size and independent selection in each sampling stratum. In the second stage, a fixed number of households per cluster were selected randomly from the household listing. Information obtained from the women’s or caregiver’s questionnaires included vaccination status and maternal and child demographic characteristics. Interviewers asked mothers to present the vaccination cards to obtain vaccination dates. In the absence of vaccination cards, such mothers were asked to recall the vaccinations received by their children. A total of 355,327 mothers of children were interviewed. In the present study, a total of 206,357 children of 12–23 months of age were included (Figure 1).

Therefore, for the study sample selection and data collection from the DHS dataset, we included all children aged 12–23 months, excluding those younger than 12 months and those older than 23 months, and a total of 206,357 were analyzed in this study. The total number of children from each country ranged from 994 in Liberia to 29,488 in Afghanistan with their respective survey years (Table 1).

**Table 1:** Study participants interviewed in each country and their respective survey year.

Country	Capital city	Year of survey	Frequency	Percent (%)
Afghanistan	Kabul	2015	29,488	14.06
Angola	Luanda	2015/16	2,500	1.18
Bangladesh	Dhaka	2017/18	1,307	0.62
Burkina Faso	Ouagadougou	2021	2,216	1.05
Benin	Porto novo	2017/ 18	2,366	1.12
Bolivia	La Paz	2008	7,869	3.73
Burundi	Bujumbura	2016/ 17	2,588	1.23
The Demographic Republic of the Congo	Kinshasa	2013/14	16,269	7.88
Côte d’Ivoire	Yamoussoukro	2021	6,679	3.18
Cameroon	Yaoundé	2018	1,595	0.76
Ethiopia	Addis Ababa	2016	1,898	0.9
Ghana	Accra	2022	5,463	2.59
Gambia	Banjul	2019/ 20	1,448	0.69
Guinea	Conakry	2018	1,365	0.65
Honduras	Tegucigalpa	2011/12	10,185	4.83
Haiti	Port-au-Prince	2016/17	1,181	0.56
Indonesia	Jakarta	2017	2,767	1.32
Kenya	Nairobi	2022	3,559	1.69
Cambodia	Phnom Penh	2021/22	1,646	0.78
Comoros	Moroni	2012	2,599	1.23
Kyrgyz Republic	Bishkek	2012	4,216	2.01
Liberia	Monrovia	2019/ 20	994	0.47
Lesotho	Maseru	2014	2,717	1.29
Morocco	Rabat	2003/04	5,850	2.78
Madagascar	Antananarivo	2021	2,248	1.07
Mali	Bamako	2018	1,765	0.84
Myanmar	Naypyidaw	2015/16	4,323	2.05
Mauritania	Nouakchott	2019/ 21	1,632	0.77
Malawi	Lilongwe	2015/ 16	3,012	1.43
Mozambique	Maputo	2022/23	4, 229	3.64
Nigeria	Abuja	2018	5,899	2.8
Niger	Niamey	2017	10,011	4.74
Nepal	Kathmandu	2022	984	0.47
Philippines	Manila	2022	1,578	0.75
Pakistan	Islamabad	2017/18	2,306	1.09
Rwanda	Kigali	2019/ 20	1,557	0.74
Sierra Leone	Freetown	2019	1,803	0.85
Senegal	Dakar	2010/11	10,632	5.04
Eswatini	Mbabane	2006/07	2,389	1.13
Chad	Ndjamena	2014/15	15, 478	7.59
Togo	Lome	2013/14	6,363	3.02
Tajikistan	Dushanbe	2017	1,242	0.59
Timor-Leste	Dili	2016	1,187	0.56
Tanzania	Dar es Salaam	2022	1,920	0.91
Uganda	Kampala	2016	2,809	1.33
Uzbekistan	Tashkent	1996	1,249	0.59
Zambia	Lusaka	2018	1,848	0.88
Zimbabwe	Harare	2015	1,135	0.54
	Total		206,357	100

### Outcome variable

Basic vaccination completeness was assessed using a composite outcome of eight doses of four vaccines for which DHS data were collected. The vaccines include Bacillus Calmette-Guérin (BCG) (one dose), polio vaccine (three doses), diphtheria-tetanus-pertussis-containing vaccines (DTP) (three doses), and measles-containing vaccines (MCV) (one dose) according to WHO guidelines (15). The DTP-containing vaccine is currently used as a pentavalent vaccine that prevents diphtheria, pertussis, tetanus (DTP), hepatitis B (HepB), and Haemophilus influenza type b (Hib) received in three doses, typically at 6, 10, and 14 weeks after birth based on DHS guidelines.

Children who received all eight vaccine doses were categorized as completely vaccinated, and those who received less than eight doses were defined as incompletely vaccinated.

### Independent variables

The following determinant variables, such as child characteristics, maternal characteristics, and demographic characteristics, were considered in the study.

Birth order of children was grouped into <4th order and ≥ 4th order (16).

Birth weight: The weight of the child at birth was grouped into three size categories: large, average, and small (17).

Level of Education was defined as no education, primary education, secondary education, or higher (16).

Wealth index was originally presented in five quintiles based on the DHS data of each country, which were derived from the measurements of ownership of household items such as car, radio, and television and dwelling features such as toilet facilities, water source, and type of roofing/floor. This mode of measurement has been used by the World Bank to categorize households into poverty levels based on principal components analysis (18). For easy interpretation, we reclassified the wealth index into three categories (poor, middle, and rich).

Marital status was grouped into unmarried, married, and separated/widowed/divorced, whereas maternal occupation was classified as not working and working.

Media exposure refers to the frequency of exposure to a newspaper, radio, television watching, and internet use. Those exposed to any of the four media access (at least once a week) were defined as having media exposure, and others were considered as not having media exposure (19).

Antenatal care attendance was defined as making at least one antenatal clinic visit during the pregnancy of the index child and categorized as attended and never attended (16).

### Data quality control

The DHS data across all resource-limited nations was comparable. The DHS guideline provided a precise definition of the missing values. Since the DHS survey is a cross-sectional study, the variables were removed from further analysis if the missing value in the explanatory variables was greater than 5%. To ensure the data quality, the data extractions were carried out by public health professionals who have experience with DHS data. The children’s record (KR) file was cleaned up before the data were extracted using standard DHS procedures. Data consistency for each of the 48 countries has been checked before being appended.

Standardized interview questions were used to measure each variable uniformly in the DHS. To compile conveniently, they recoded uniformly with other countries during data management. The primary investigator and supervisor have assessed the data daily for consistency and completeness. The extent of incomplete basic vaccination among women in each nation was assessed with the respective DHS reports.

### Data management and statistical analysis

Before conducting any statistical analysis, the data were weighted and carefully examined. The data extraction, recoding, and analysis were conducted using STATA V.17. A sampling weight was conducted to adjust for the non-proportional allocation of the sample to different regions and the possible differences in response rates. Hence, the actual representativeness of the survey results at both the national and regional levels was ensured. Frequencies and percentages were used to show descriptive statistics.

### Spatial analysis

For the spatial analysis, ArcGIS version 10.7 software was used, and for SaTScan analysis, SaTScan version 10.1 software was used.

### Spatial autocorrelation analysis

The spatial autocorrelation (Global Moran’s I) statistic was used to measure whether incomplete basic vaccination was dispersed, clustered, or randomly distributed in the study area (20). Moran’s I is a spatial statistic used to measure spatial autocorrelation by taking the entire data set and producing a single output value, which ranges from −1 to +1. Moran’s I values close to −1 indicate incomplete basic vaccination dispersed, whereas Moran’s I values close to +1 indicate incomplete basic vaccination clustered, and the I value of zero indicates incomplete basic vaccination distributed randomly. A statistically significant Moran’s I (*p* < 0.05) leads to rejection of the null hypothesis.

### Incremental spatial autocorrelation

Incremental spatial autocorrelation was performed to obtain the maximum peak distance where incomplete basic vaccination clustering is more pronounced. The maximum peak distance is the distance where maximum spatial autocorrelation occurs, and this was used as a distance band for hotspot analysis.

### Hotspot analysis (Getis-OrdGi* statistic)

Getis-OrdGi* statistics was performed to measure how spatial autocorrelation varies over the study location by calculating GI* statistic for each area. Z-score was computed to determine the significant hotspot and significant coldspot areas of incomplete basic vaccination. The statistical output with high GI* was used to indicate “hotspot,” whereas low GI* indicates “coldspot” per proportion of incomplete basic vaccination among children aged 12–23 months.

### Spatial scan (sat scan) statistical analysis

In the spatial scan statistical analysis, a Bernoulli-based model was employed to identify statistically significant spatial clusters of children with incomplete basic vaccination using Kuldorff’s SaTScan version 10.1 software. Children with incomplete basic vaccination were taken as cases, and those with complete basic vaccination were considered as controls to fit the Bernoulli model. The number of cases in each location had a Bernoulli distribution, and the model required data for cases, controls, and geographic coordinates. A Likelihood Ratio (LR) test statistic and the *p*-value were applied to determine whether the number of observed women with incomplete basic vaccination within the possible cluster was considerably higher than anticipated. The scanning window with the highest likelihood was the cluster that was most likely performing well (primary). For each identified cluster, the Log-Likelihood Ratio (LLR) test statistic with its *p*-value, the Relative Risk (RR), the location radius, population, and cases were reported.

### Model building for multilevel analysis

We fitted a multilevel mixed-effects binary logistic regression model, since the standard binary logistic regression model’s independent assumption is violated by the hierarchal nature of DHS data. To choose the appropriate model for the study, four models were fitted. The first is the null model (Model I), which contains no exposure variables and is used to check the variability of incomplete basic vaccination in the community. Then, Model II and Model III multilevel models contained individual-level and community-level variables, respectively. In the last model (Model IV), both individual- and community-level variables were fitted simultaneously with the outcome variable. Model comparison was performed using deviance, and the model with the lowest deviance was selected as the best-fitted model. Both bivariable and multivariable multilevel logistic regression were performed to identify the determinants of incomplete basic vaccination. Finally, to determine the association of predictors, variables with a *p*-value of ≤0.2 at bivariable analysis were selected for multivariable analysis, and in multivariable analysis, variables with a p-value less than 0.05 were considered significantly associated factors.

### Parameter estimation method

The fixed effects were used to estimate the association between the likelihood of the magnitude of incomplete basic vaccination and explanatory variables both at individual and community levels. Associations between dependent and independent variables were assessed, and their strengths were presented using Adjusted Odds Ratio (AOR) and 95% confidence intervals with a *p*-value of < 0.05.

Log ( $\frac{\pi ij}{1-\pi ij}$ ) = βo + β1xij + β2xij + …uj + eij.

Where,

*πij:* the probability of incomplete basic vaccination.

1 − *πij*: the probability of complete basic vaccination.

β1xij are individual- and community-level variables for the ith individual in group j, respectively. The ß’s are fixed coefficients indicating that a unit increase in X can cause a ß unit increase in the probability of incomplete basic vaccination. While ß_0_ is the intercept, the effect on incomplete basic vaccination is when the effect of all explanatory variables is absent. The uj shows the random effect (effect of the community on the children’s incomplete basic vaccination) for the jth community. The clustered data nature and the within and between community variations were taken into account, assuming each community has a different intercept (*β*_0_) and fixed coefficient (β) (21, 22).

Random effects were estimated by the median odds ratio (MOR), Intra Class Correlation Coefficient (ICC), and Proportional Change in Variance (PCV).

The MOR is defined as the median value of the odds ratio between the area at the highest risk and the area at the lowest risk when randomly picking out two clusters.

MOR = exp.[√(2 × VA) × 0.6745], or MOR = e^0.95√VA^ where VA is the area level variance (23, 24).

The PCV reveals the variation in the prevalence of incomplete basic vaccination among children 12–23 months explained by factors. The PCV is calculated as PCV= $\frac{\mathrm{Vnull}-VA\;}{\mathrm{Vnull}}$ ∗100 where V_null_ = variance of the initial model and VA = variance of the model with more terms.

The ICC, which reveals the variation of incomplete basic vaccination between clusters, is calculated as ICC= $\frac{VA}{VA+3.29}$ ∗100%, where VA = area/cluster level variance (25, 26).

## Ethical consideration

We obtained permission from the DHS Programme to download and utilize the data for this study from http://www.dhsprogram.com. No identifying information about respondents, households, or sample communities was disclosed. The data files do not contain names or addresses of individuals; geographic identifiers only represent regional levels, encompassing broad geographic areas, including multiple states or provinces. Each primary sampling unit (EA) in the data is identified solely by a number without any associated labels indicating names or specific locations. The data collection adhered to the principles outlined in the Declaration of Helsinki, following the receipt of written informed consent from study participants.

## Results

### Background characteristics of study participants

A total of 202,029 weighted children aged 12–23 months were included in this study. Almost half (48.31% of children’s mothers) were between the ages of 25 and 34 years, with a median age of 28 (IQR: 15) years. Almost 90% of mothers of children were married. Of the total of our study participants, nearly 66% of them had media exposure, and 85% of mothers attended antenatal care. Regarding children’s birth weight, nearly 81% of them were large and above.

The percentage of women concerned with their wealth status and poor income was 43%. Less than half of the women have an occupation (working).

According to community-level factors, 68% of the study participants are from rural residences. In this study, 57 % of children in resource-limited countries were from low-income countries (Table 2).

**Table 2:** Background characteristics of mothers and children in resources-limited countries DHS, 2024.

Variables	Categories	Frequency		Weighted percentage (%)
		Unweighted	Weighted	
Maternal age	15–24 years	63,803	62,536.461	30.23
	25–34 years	101,431	101,593.32	48.31
	35–49 years	45,786	44,388.947	21.46
Sex of child	Male	107,338	104,925.34	50.72
	Female	103,682	101,945.64	49.29
Sex of household head	Male	178,559	175,079.88	83.60
	Female	34,771	34,348.808	16.40
Maternal marital status	Single	8,604	8,409.7987	4.07
	Married	191,261	187,348.57	90.56
	Widowed/Divorced/separated	11,155	11,112.611	5.37
Maternal Education	No education	93,658	88,539.477	42.80
	Primary education	59,460	59,227.424	28.73
	Secondary and higher	57,902	59,104.079	28.57
Husband’s Education	No education	74,453	70,883.081	36.58
	Primary education	50,235	49,498.675	25.55
	Secondary and higher	72,961	73,371.9717	37.87
Media exposure	Yes	43,871	43,324.189	65.94
	No	22,862	22,373.474	34.06
Wealth Status	Poor	96,959	88,469.118	43.04
	Middle	42,511	41,662.87	20.27
	Rich	70,300	75,429.848	36.69
Maternal Occupation	Working	94,631	94,069.34	45.56
	Not working	115,969	112,401.07	54.44
ANC visit	Never attended	25,934	23,817.01	15.16
	Attended	133,878	133,328.27	84.84
Birth size	Large	62,053	61,373.74	30.66
	Very large	103,732	101,170.32	50.53
	Small	38,491	37,660.531	18.81
Birth order	<4	124,338	123,594.11	59.74
	> = 4	86,682	83,276.874	40.26
Place of delivery	Home	78,310	72,899.623	35.88
	Health facility	129,084	130,284.22	64.12
Mode of delivery	Cesarean delivery	13,413	14,470.038	7.13
	Vaginal delivery	193,880	188,601.99	92.87
Health insurance coverage	Yes	17,299	16,573.061	9.09
	No	168,388	165,668.13	90.91
Distance from health facility	Big problem	91,155	86,262.679	44.56
	Not a big problem	106,183	107,334.25	55.44
Residence	Urban	64,195	65,283.4924	31.56
	Rural	146,825	141,587.49	68.44
Country level income	Low income	120,210	119,358.95	57.70
	Lower middle income	90,810	87,512.035	42.30

### Components of basic vaccination

Globally, in resource-limited countries, Bacillus Calmette-Guérin (BCG) is the most offered component of basic vaccination (81.7%), ranging from 50.51% in Chad to 99.23% in Rwanda. In total, 65% of children received the measles-containing vaccine (MCV) in resource-limited countries, ranging from 37.66% in Guinea to 97.75% in Rwanda. The least offered components of basic vaccination were the provision of diphtheria, pertussis, and tetanus (DTP) (63.21%). It varied from the lowest of 24.64% in Chad to the highest of 98.91% in Rwanda (Table 3).

**Table 3:** Components of basic vaccination to be given for children aged 12–23 months.

Country	Bacillus Calmette-Guérin (%)	Diphtheria, pertussis, tetanus (%)	Polio (%)	Measles containing vaccine (%)
	Yes	Yes	Yes	Yes
Afghanistan	63.45	41.96	53.74	46.15
Angola	70.88	38.56	39.92	52.64
Bangladesh	97.94	95.26	94.19	88.39
Burkina Faso	97.29	88.85	81.49	85.88
Benin	87.07	72.74	65.59	67.54
Bolivia	97.41	77.28	77.57	70.74
Burundi	97.91	96.59	92.08	93.39
The Democratic Republic of the Congo	76.07	51.02	55.21	60.73
Côte d’Ivoire	79.59	56.36	61.43	56.22
Cameroon	86.14	70.91	66.52	64.95
Ethiopia	70.28	55.95	60.54	58.17
Ghana	94.84	77.02	73.15	74.53
Gambia	98.48	93.44	90.88	91.92
Guinea	71.36	37.44	37.22	37.66
Honduras	98.42	83.79	83.97	75.64
Haiti	81.88	56.14	56.14	61.98
Indonesia	89.84	75.52	72.10	78.896
Kenya	95.95	86.57	74.91	84.74
Cambodia	93.13	81.23	83.90	79.95
Comoros	83.69	61.83	55.37	62.64
Kyrgyz Republic	98.80	80.19	73.63	78.55
Liberia	91.45	68.11	61.67	71.93
Lesotho	94.44	76.89	64.85	74.83
Morocco	96.64	85.78	86.24	77.05
Madagascar	76.51	67.62	58.27	63.35
Mali	80.74	66.23	51.33	66.52
Myanmar	85.34	58.31	63.46	69.60
Mauritania	90.99	74.02	47.98	76.78
Malawi	97.51	93.26	82.60	91.69
Mozambique	89.10	54.81	54.46	53.58
Nigeria	66.82	49.78	46.31	53.67
Niger	78.40	59.35	67.99	59.46
Nepal	95.63	90.04	86.69	89.33
Philippines	84.66	77.25	74.90	76.81
Pakistan	83.95	70.86	83.78	68.13
Rwanda	99.23	98.91	97.56	97.75
Sierra Leone	96.78	78.87	72.38	76.21
Senegal	91.72	73.40	59.72	71.58
Eswatini	95.56	83.72	73.63	77.73
Chad	50.51	24.64	42.19	47.20
Togo	92.89	76.44	63.06	66.85
Tajikistan	94.61	82.61	83.09	80.43
Timor-Leste	82.39	63.86	55.01	72.28
Tanzania	92.55	91.30	62.45	86.98
Uganda	96.65	79.71	66.99	80.88
Uzbekistan	97.04	77.34	80.4	70.16
Zambia	97.35	92.05	81.82	90.85
Zimbabwe	91.54	85.46	84.23	84.49
All countries (globally)	81.71%	63.21%	63.96%	65.02%

### Pooled prevalence of incomplete basic vaccination among children in resource-limited countries

**Figure 2:**
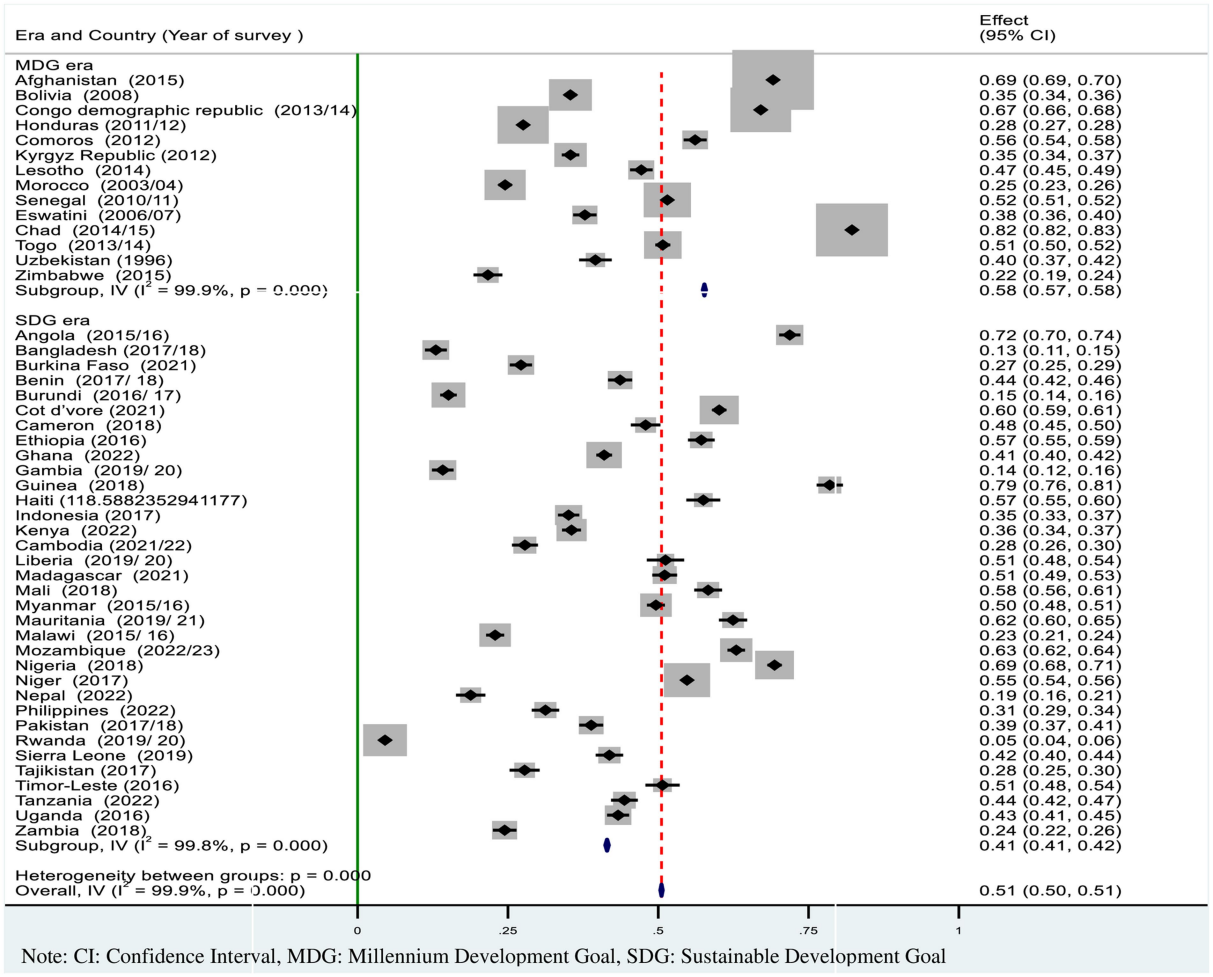
Forest plot showed the pooled prevalence of incomplete basic vaccination among children 12–23 months in resource-limited countries.

As presented in Figure 2, the overall pooled prevalence of incomplete basic vaccination was 51% (95% CI: 50, 51) among children aged 12–23 months in resource-limited countries. In the SDG era, nearly 41% of children had incomplete basic vaccinations globally. It covers a range of 5% in Rwanda to 82% in Chad (Figure 2).

### Spatial regression analysis of incomplete basic vaccination among children in resource-limited countries

#### Spatial distribution of incomplete basic vaccination

**Figure 3:**
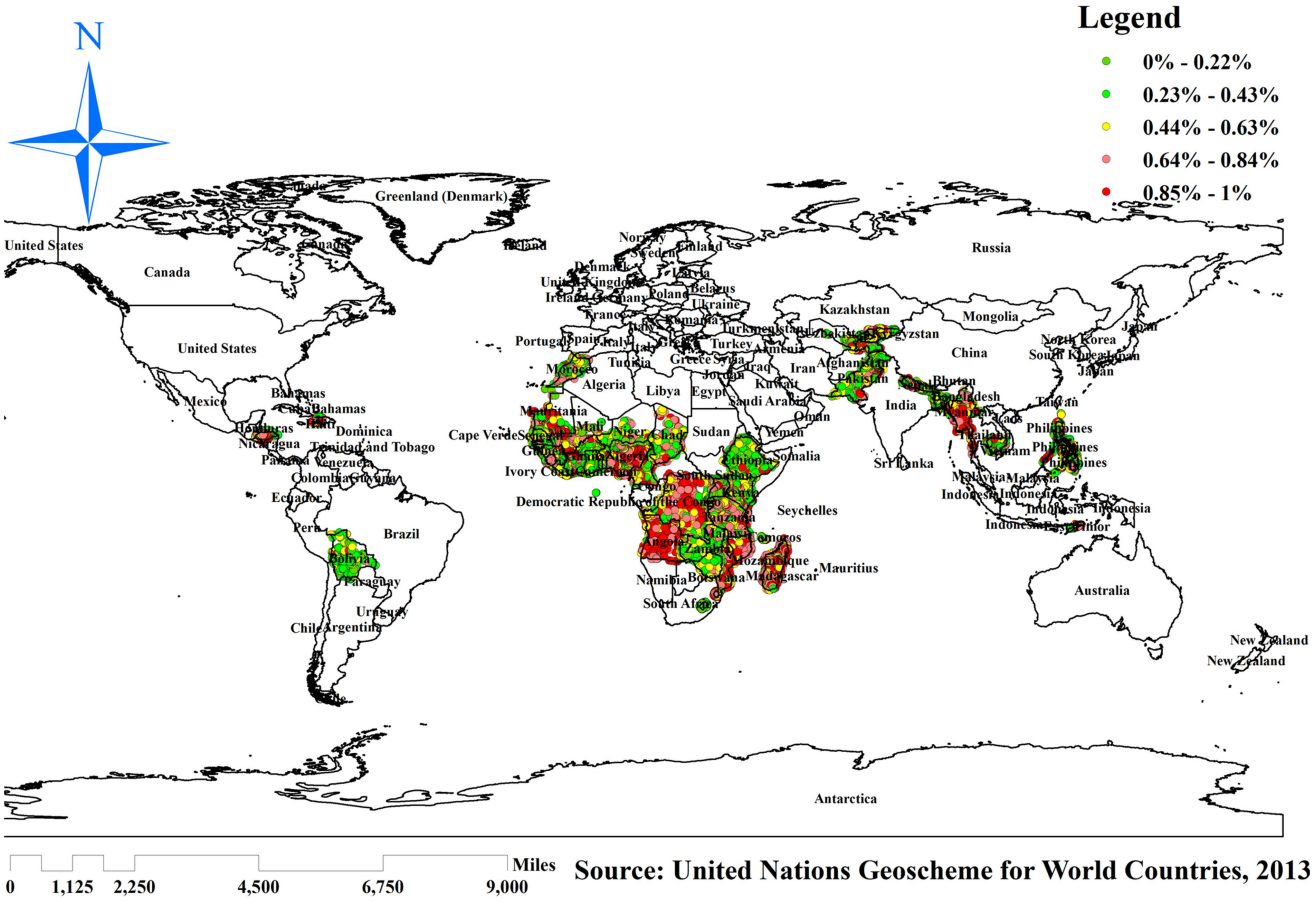
Spatial distribution of incomplete basic vaccination among children in resource-limited countries. Source: World map created based on United Nations Geoscheme for World Countries, 2013 data, using map elements from GADM, published under GADM license.

The highest proportion of incomplete basic vaccination was spatially clustered in sub-Saharan Africa (Angola, Nigeria, Cameroon, Chad, Madagascar, and Guinea), Haiti, Afghanistan, the Philippines, and Myanmar (Figure 3).

#### Spatial autocorrelation analysis of incomplete basic vaccination

**Figure 4:**
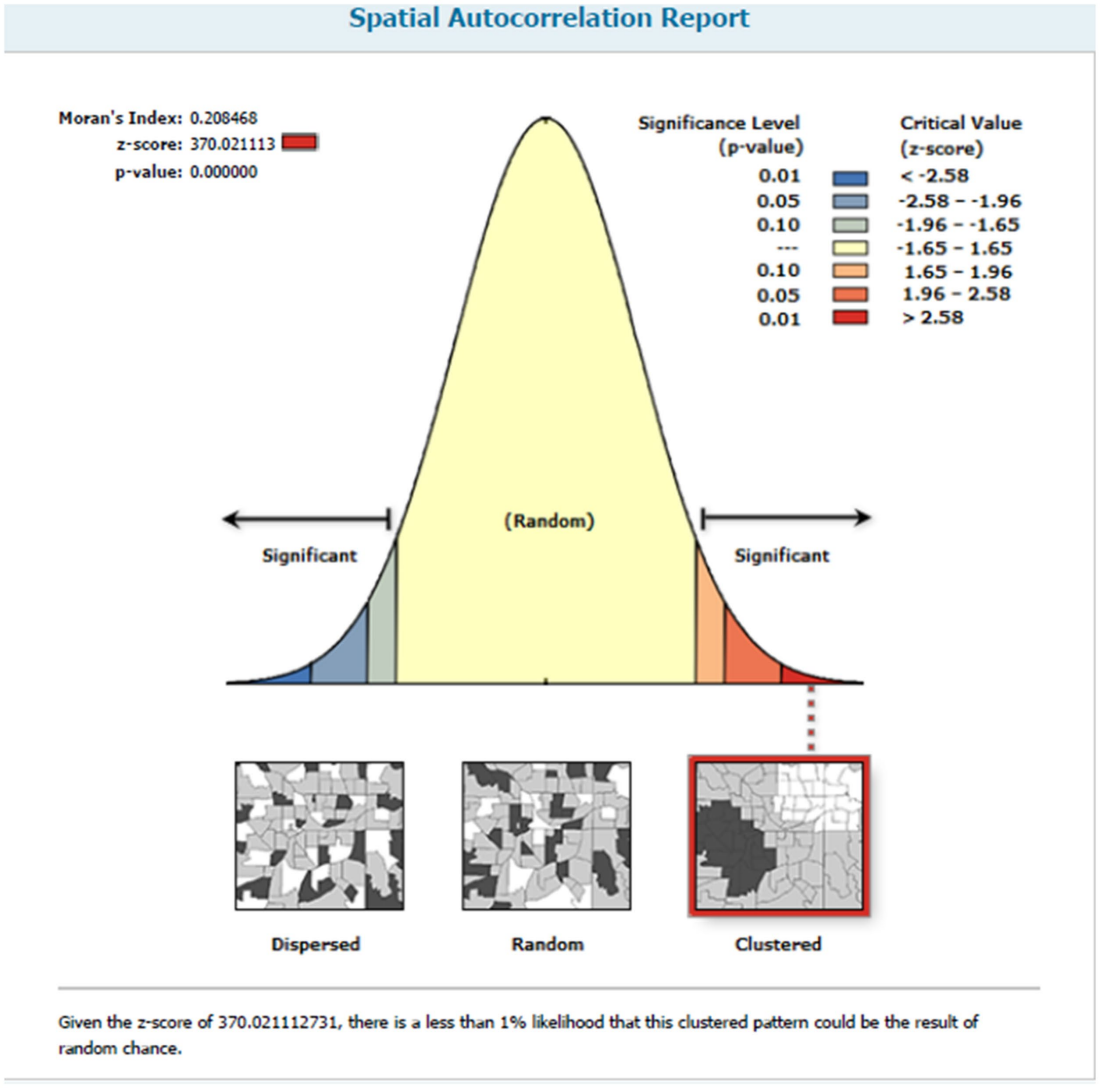
Spatial autocorrelation report of incomplete basic vaccination among children in resource-limited countries.

This study revealed that the spatial distribution of incomplete basic vaccination was found to be spatially clustered in resource-limited countries with Global Moran’s I = 0.208468 (*p* < 0.001) (Figure 4).

### Hotspot (Getis-Ord Gistatistic) analysis of incomplete basic vaccination

**Figure 5:**
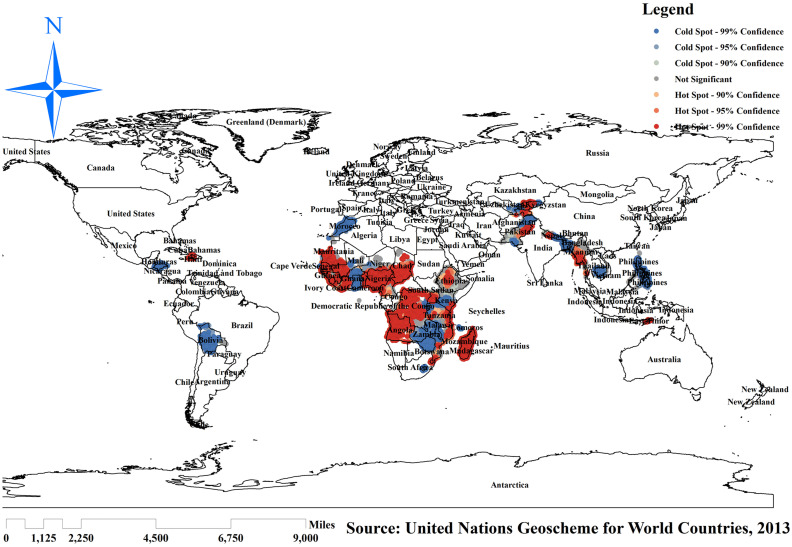
Hotspot analysis of incomplete basic vaccination among children in resource-limited countries. Source: World map created based on United Nations Geoscheme for World Countries, 2013 data, using map elements from GADM, published under GADM license.

Hotspot analysis was performed to identify areas with high levels of incomplete basic vaccination among reproductive women in resource-limited countries globally. The red color indicates significant areas with a high number of children with incomplete basic vaccination in resource-limited countries. It is found in Guinea, Ghana, Mauritania, Niger, Nigeria, Chad, Cameroon, Angola, Central Ethiopia, Madagascar, Haiti, Myanmar, Afghanistan, and Pakistan. In contrast, the green color indicates areas of having complete basic vaccination and is observed in Bolivia, Morocco, Mali, Kenya, Zambia, Nepal, and the Philippines (Figure 5).

#### Spatial SaTScan statistics analysis

The spatial scan statistics showed 10,347 significant clusters, of which 2,875 were most likely/primary, 948 were secondary, and 920 were tertiary clusters. The most likely clusters were located in Nigeria, Chad, Cameroon, and Niger, which were centered at (2.028929 N, 15.135990 E)/1425.16 km radius, Log-Likelihood Ratio (LLR) of 3519.48, and Relative Risk (RR) of 1.38 at *p*-value <0. 001, which imply that children with a spatial window have 1.38 times higher likelihood of having an incomplete basic vaccination as compared to children outside of the window. The secondary clusters’ spatial window was located in Angola, the Democratic Republic of the Congo, Madagascar, and Western Parts of Nigeria and Madagascar that was centered at (10.160391 N, 13.350180 E) with 567.24 km radius, Log-Likelihood Ratio (LLR) of 1835.987, and Relative Risk (RR) of 1.34 at a *p*-value of 0.001, which means children within the spatial window are 1.34 times more likely to have incomplete basic vaccination than children outside of the window.

**Figure 6:**
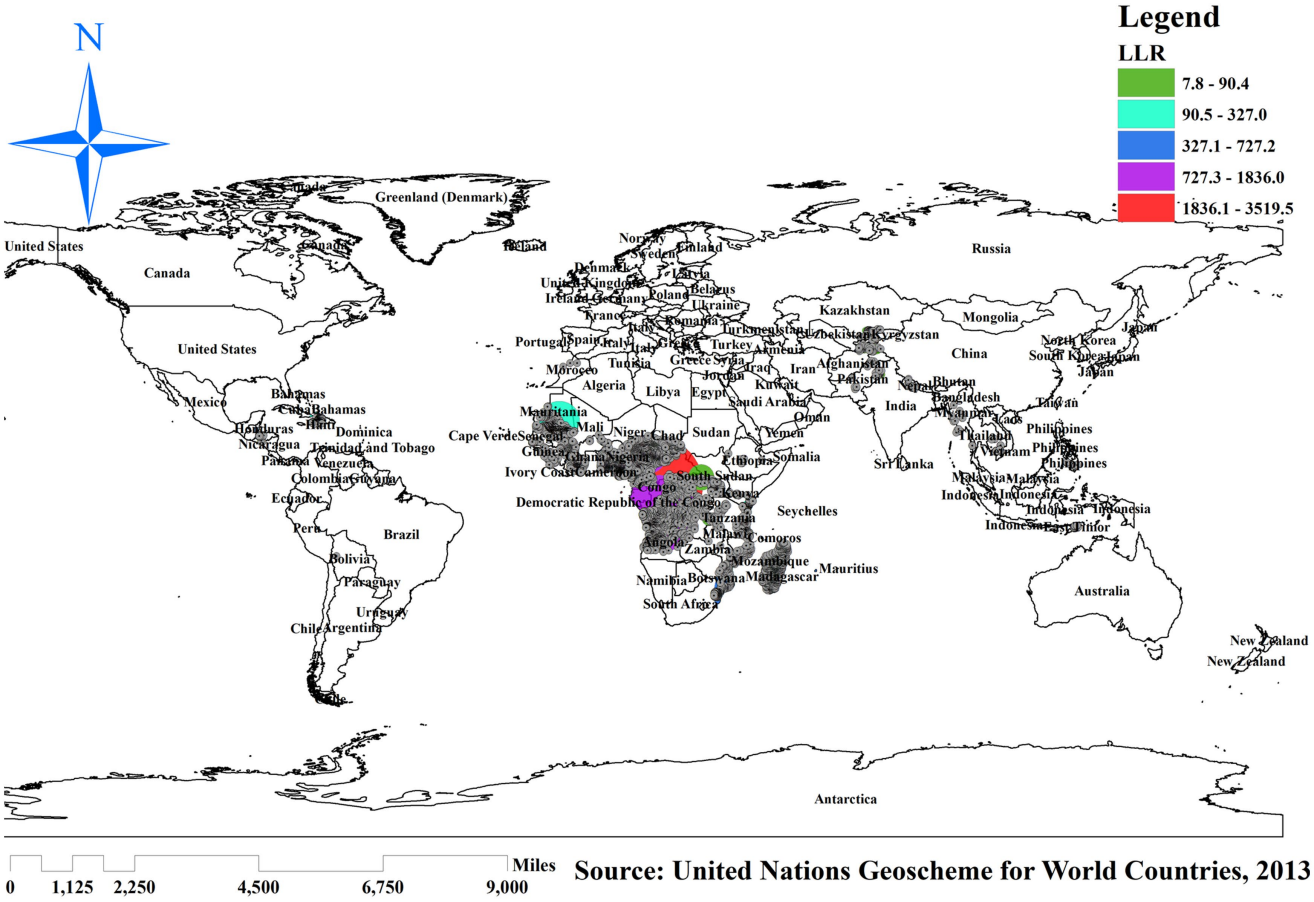
SaTScan analysis of incomplete basic vaccination among children in resource-limited countries. Source: World map created based on United Nations Geoscheme for World Countries, 2013 data, using map elements from GADM, published under GADM license.

The tertiary clusters’ spatial window was located in Zambia, Guinea, Botswana, Madagascar, and Myanmar, centered at (5.349280 S, 16.644730 E) with a 1120.38 km radius, a Log-Likelihood Ratio (LLR) of 1283.89, and a Relative Risk (RR) of 1.32 at *p*-value 0. 001. This indicates that children within the spatial window have a 1.32 times higher likelihood of incomplete basic vaccination than children outside the window (Figure 6).

#### Parameters and model comparison for multilevel analysis

The ICC in model-one (null model) was 0.347, which indicates that 34.7% of the variation of incomplete basic vaccination among children was attributed to cluster difference, while 65.3% was attributed to individual woman and child factors.

The MOR value was 5.252 in the null model, indicating that the odds of incomplete basic vaccination among children differed across clusters.

Regarding the PCV value, 0.514 in the final model points out that 51.4% of the variation of incomplete basic vaccination among children aged 12–23 months was explained by the last model. In regard to the model fitness, deviance was checked, and the model with the lowest deviance was the best model (Model-IV = 44, 939.572), selected for interpretation (Table 4).

**Table 4:** Parameters and model fit statistics for multilevel regression analysis models.

Parameters	Null model	Model II	Model III	Model VI
Cluster level variance	1.746	1.142	0.972	0.849
ICC	0.347*	0.258*	0.228*	0.205*
MOR	5.252	2.803	2.404	2.152
PCV	Reference	0.346	0.443	0.514
Model fitness				
Deviance	248, 244.28	52, 852.922	60,387.258	44, 939.572
Mean VIF	-	1.60	1.21	1.65

ICC: Inter cluster correlation coefficient; MOR: Median odds ratio; PCV: Proportional change in variance; VIF: Variance inflation factors. ^*^P-value < 0.05.

#### Factors associated with incomplete basic vaccination among children in resource-limited countries

Both individual- and community-level factors with a *p*-value <0.2 in the bivariable analysis were selected for multilevel analysis. Based on the final model analysis from individual-level variables, age, marital status, maternal education, husband’s education, maternal occupation, media exposure, wealth index, ANC visits, birth order, place of delivery, mode of delivery, health insurance coverage, perception of distance from health facility, place of residence, community media exposure, community education, and country-level income status were statistically significant associated with incomplete basic vaccination (*p* ≤ 0.05).

The odds of incomplete basic vaccination were 1.42 (AOR = 1.42, 95%CI: 1.28, 1.56) among mothers aged 15–24 and 1.12 (AOR = 1.12, 95% CI: 1.04, 1.21) times higher among mothers aged 25–34 as compared to women aged 35–49 years. The odds of incomplete basic vaccination is 1.25 times (AOR = 1.25, 95% CI: 1.15, 1.85) among unmarried mothers. For mothers with no education and primary education, the odds of incomplete basic vaccination were 1.85 times (AOR = 1.85, 95% CI: 1.22, 1.49) and 1.11 times (AOR = 1.11, 95% CI: 1.03, 1.21) higher than mothers with secondary and above formal education, respectively. For husbands with no formal education, the odds of incomplete basic vaccination were 1.17 times (AOR = 1.17, 95% CI: 1.07, 1.28) higher than those who had secondary and higher education. The odds of incomplete basic vaccination among mothers with no media exposure was 1.14 times (AOR = 1.14, 95%CI: 1.06, 1.22) higher than those with media exposure.

The odds of incomplete basic vaccination among mothers of poor-income status were 1.22 times higher (AOR = 1.22, 95%CI: 1.12, 1.84), and among those of middle income, the odds were 1.18 times (AOR = 1.18, 95%CI: 1.04, 1.28) higher than rich individuals.

The odds of incomplete basic vaccination for non-worker mothers were higher by 1.28 times (AOR = 1.28, 95% CI: 1.16, 1.80) than for mothers who are working. The odds of incomplete basic vaccination were 2.97 times (AOR = 2.97, 95%CI: 2.67, 8.80) higher among women who had no ANC visits than their counterparts.

The odds of incomplete basic vaccination among children with birth order four and above were 1.28 times (AOR = 1.28, 95%CI: 1.15, 1.84) higher than children who were under four.

Higher odds of incomplete basic vaccination among mothers who reported home delivery as compared to mothers who delivered at the health facility (AOR = 2.46: 95%CI: 2.28, 2.64), and cesarean mode of delivery was highly related to incomplete basic vaccination (AOR = 1.17, 95%CI: 1.05, 1.31). Reproductive-age mothers with no health insurance coverage were 1.78 times (AOR = 1.78, 95%CI: 1.59, 1.99) higher incomplete basic vaccination than mothers with health insurance coverage.

From community-level variables, place of residence, community media exposure, community education, and country income status were significantly associated with incomplete basic vaccination. A mother living in a rural area had 1.43 times (AOR = 1.43, 95%CI: 1.30, 1.58) higher odds of incomplete basic vaccination than a mother living in an urban area. Moreover, there are high odds of incomplete basic vaccination observed among mothers with low community media exposure and low community education (AOR = 1.19, 95%CI: 1.09, 1.31) and (AOR = 1.48, 95%CI: 1.35, 1.62), respectively. The odds of incomplete basic vaccination among mothers in lower middle-income countries were 1.58 times (AOR = 1.58, 95% CI: 1.45, 1.71) higher than mothers from low-income countries (Table 5).

**Table 5:** Multilevel analysis of factors associated with incomplete basic vaccination among children 12–23 months in resource-limited countries: based on the recent DHS data.

Variable	Category	Null model	Model II	Model III	Model IV
		AOR (95% CI)	AOR (95% CI)	AOR (95% CI)	AOR (95% CI)
Maternal age	15–24 years	-	1.43 (1.31, 1.57)***	-	1.42 (1.28, 1.56)***
	25–34 years	-	1.11 (1.04, 1.19)***	-	1.12 (1.04, 1.21)***
	35–49 years	-	1	-	1
Sex of child	Male	-	1	-	1
	Female	-	0.98 (0.94, 1.03)	-	0.99 (0.94, 1.04)
Sex of household	Male	-	0.93 (0.87, 1.02)	-	0.89 (0.82, 0.96)
	Female	-	1	-	1
Maternal marital status	Married	-	1	-	1
	Unmarried	-	1.21 (1.13,1.30)***	-	1.25 (1.15, 1.35)***
Maternal education	No education	-	1.45 (1.33, 1.58)***	-	1.35 (1.22, 1.49)***
	Primary education	-	1.06 (0.99, 1.15)	-	1.11 (1.03, 1.21)**
	Secondary and higher	-	1	-	1
Husband’s education	No education	-	1.23 (1.14, 1.34)***	-	1.17 (1.07, 1.28)***
	Primary education	-	0.89 (0.83, 0.96)*	-	0.94 (0.87, 1.02)
	Secondary and higher	-	1	-	1
Media exposure	Yes	-	1	-	1
	No		1.16 (1.09, 1.24)***	-	1.14 (1.06, 1.22)***
Wealth status	Poor	-	1.10 (1.02, 1.19)**	-	1.22 (1.12, 1.34)***
	Middle	-	1.04 (0.971.13)	-	1.13 (1.04, 1.23)**
	Rich	-	1	-	1
Maternal Occupation	Working	-	1	-	1
	Not working	-	1.18 (1.12, 1.25)***	-	1.23 (1.16, 1.30)***
ANC visit	Never attended	-	2.92 (2.65,3.21)***	-	2.97 (2.67, 3.30)***
	Attended	-	1	-	1
Birth size	Large	-	1.02 (0.94, 1.11)	-	0.98 (0.90, 1.07)
	Very large	-	1.00 (0.93, 1.08)	-	0.97 (0.89, 1.05)
	Small	-	1	-	1
Birth order	<4	-	1	-	1
	> = 4	-	1.24 (1.16, 1.33)***	-	1.23 (1.15, 1.34)***
Place of delivery	Home	-	2.44 (2.28, 2.61)***	-	2.46 (2.28, 2.64)***
	Health facility	-	1	-	1
Mode of delivery	Cesarean	-	1.09 (0.99, 1.21)	-	1.17 (1.05, 1.31)**
	Vaginal	-	1	-	1
Health insurance coverage	Yes	-	1	-	1
	No	-	2.04 (1.84, 2.27)***	-	1.78 (1.59, 1.99)***
Distance from health facility	Big problem	-	-	1.22 (1.15, 1.28)***	1.05 (0.98, 1.12)
	Not big problem	-	-	1	1
Residence	Urban	-	-	1	1
	Rural	-	-	1.22 (1.12, 1.32)***	1.43 (1.30, 1.58)***
Community media exposure	High	-	-	1	1
	Low	-	-	1.71 (1.57, 1.87)***	1.19 (1.09, 1.31)***
Community education	High	-	-	1	1
	Low	-	-	2.55 (2.35, 2.77)***	1.48 (1.35, 1.62)***
Country level income	Low income	-	-	1	1
	Lower middle income	-	-	1.69 (1.57, 1.82)***	1.58 (1.45, 1.71)***

AOR, adjusted odds ratio; CI, confidence interval. Values expressed in Table 5. ^*^p-value < 0.05. ^**^p value < 0.01. ^***^p value < 0.001.

## Discussion

Incomplete basic vaccination is a global problem among children aged 12–23 months, even though it was considered one of the strategic plans of SDG to maximize immunization coverage by 2030 (27). The crucial approach to addressing the problem’s impact is identifying and minimizing the preventable underlying factors. Consequently, the objective of this study is to analyze the geographical distribution and associated factors of incomplete basic vaccination among children in resource-limited countries worldwide.

In our study, the pooled magnitude of incomplete basic vaccination among children aged 12–23 months was 51% (95%CI: 50–51%). This finding was higher than those reported in East Africa (30.79%) (17), in Africa (35.5%) (11), in South Africa (40.8%) (16), in Indonesia (40%) (28), and in Ethiopia (30%) (29), and lower than a study conducted in Nigeria (69.6%) (30). The possible reasons for the discrepancy in the magnitude of incomplete basic vaccination are maternal and geographical factors like the province of residence (16) and inequalities in children’s immunization programs (17). Another reason for incomplete basic vaccination is mothers’ cultural misconceptions regarding vaccination, and the associated adverse effects of vaccinations were the main causes of incomplete basic vaccination and reduction in immunization coverage despite an increase in vaccine-preventable disease outbreaks (31).

The spatial regression analysis found that the spatial distribution of incomplete basic vaccination across resource-limited countries was varied. Significant hotspot areas of incomplete basic vaccination were detected in Guinea, Ghana, Mauritania, Niger, Nigeria, Chad, Cameroon, Angola, Central Ethiopia, Madagascar, Haiti, Myanmar, Afghanistan, and Pakistan. This might be due to the inadequacy of maternal and child healthcare services, which is related to a lack of awareness about the risk factors following incomplete basic vaccination in those resource-limited countries (32, 33). Even though these countries are rapidly transitioning in socioeconomic growth (34), this finding suggests that children are receiving inadequate and unequal immunization services worldwide. As a result, public health programmers should develop intervention strategies to decrease the prevalence of incomplete basic vaccination in these significant hotspot areas.

In our study, both maternal and child characteristics were significantly associated with incomplete basic vaccination among children in resource-limited countries. The current study revealed that mothers aged 25–34 and 35–49 years were positively associated with incomplete basic vaccination. A unit increase in age increases the odds of having incomplete basic vaccination by 1.4 and 1.12 times higher for mothers aged 15–24 and 25–34 years, compared to mothers aged 35–49 years, respectively. This finding is consistent with a study conducted in Ethiopia (35), Pakistan (36), and Bangladesh (37). The possible reason might be explained by the fact that younger caregivers do not have adequate experience in giving care to their children (38). Furthermore, older caregivers may encounter difficulties providing care for ill children, leading to a strain on household finances. Consequently, these mothers may strongly commit to preventive measures such as vaccination services (39).

Our study indicated that children of mothers who were unmarried had 1.25 times higher odds of incomplete basic vaccination than married mothers. This was supported by a study in Ethiopia (40). This is due to the fact that being unmarried increases the likelihood of never attending or missing ANC follow-up compared to a married mother. Additionally, this can be explained by the fact that married couples typically make decisions together, including those concerning their children’s healthcare services. The collaborative decision-making process probably leads to a higher likelihood of reaching a mutual agreement to ensure basic vaccination for their child timely.

Having no education was significantly associated with incomplete basic vaccination compared to individuals with secondary and higher educational status for both mother and husband. This is supported by a study conducted in Canada (41), Iraq (42), and Cameroon (43). This indicates that mothers’ level of education is crucial in ensuring that children receive the necessary vaccinations. This is likely because educated mothers and caregivers can access and understand information about vaccines, their benefits, and potential side effects. The ability to read and comprehend this information is essential in making informed decisions about the importance of starting vaccinations early and ensuring they are completed (44).

Mothers of children who have no media exposure were highly vulnerable to incomplete basic vaccination. This is congruent with a study in Cameroon (43), Ethiopia (19), and Vietnam (45). This indicates that increased media exposure enhances interpersonal communication and sharing of information regarding cultivating the desire of caregivers/mothers toward childhood basic vaccine programs (46).

The participants with poor and middle wealth were more likely to have an incomplete basic vaccination of children aged 12–23 months; this finding is supported by the findings from the secondary data analysis in Malawi (47), Bangladesh (37), and Canada (41). This may be due to a lack of money for transportation to receive the vaccination from health services. Consequently, mothers of the richest wealth status have a higher likelihood of accessing modern healthcare services for their families, particularly their children, which implies greater freedom. Conversely, children of mothers in the lowest wealth quintile are less likely to complete their children’s vaccinations due to challenges in accessing facilities, such as transportation fees (37, 48).

This study revealed that mothers who had no work were more likely to have incomplete basic vaccinations for their children. This was supported by a study in the United States (49). However, it was contradictory to the findings in Bangladesh (37). As was explained in a study (30), working mothers may have the opportunity to receive important information about childhood vaccination while performing their daily responsibilities, either at their workplace or through interactions in various social settings.

The result of our study identified that mothers of children who never attended antenatal care services were significantly associated with incomplete basic vaccination of children. Similarly, a study was conducted in Nigeria (50) and Benin (51). Participating in antenatal clinics provides mothers with essential knowledge about vaccination and other child health practices. Through personalized counseling and health education sessions at these clinics, mothers are educated about the importance of vaccination, gain confidence in the available health services, are encouraged to opt for institutional delivery, and ultimately enhance the uptake of childhood vaccinations (52). Furthermore, healthcare providers should offer pregnant women sufficient information on the importance of postnatal immunization during ANC (51).

Another factor that has a significant association with incomplete basic vaccination is birth order. Children who are within the birth order of four and above have high odds of receiving an incomplete basic vaccination. Previous studies, such as those in Southeastern Ethiopia and the United States (53, 54), also reported that first- and second-order children receive updated basic vaccinations in a timely manner. The possible reason may be that firstborn children are unique for new parents and get appropriate vaccinations on time. However, as the number of children in the household grows, limited resources may result in incomplete basic vaccination and increased childhood illness.

Similarly, mothers who gave birth at home were 1.28 times more likely to have incomplete basic vaccination. In agreement with our findings, studies in East African countries also identified a significant association between home delivery and incomplete childhood vaccination (55). This is due to the fact that mothers who deliver at health facilities have the chance for their children to start childhood vaccinations such as BCG and polio since birth. Therefore, women delivering in healthcare facilities are more likely to receive comprehensive education from healthcare providers regarding the significance of childhood vaccinations, enhancing their trust in utilizing preventive healthcare services.

Accordingly, mothers of children without health insurance coverage were significantly associated with incomplete basic vaccination. Some studies (44, 56, 57) report findings similar to those of the current study. This might be due to the fact that having health insurance coverage encourages mothers of children to have continuous consultations about childhood immunization programs.

In our study, children from rural areas had higher odds of having incomplete basic vaccination than those from urban areas. Similarly, studies conducted in Nigeria (58), Africa (11), and Ethiopia (29) reported that children from urban areas receive more complete vaccination than children residing in rural areas. This could be due to mothers of children from rural areas being less knowledgeable about vaccination programs since they are far from health facilities, media access, and limited/no health professional contact (11).

Low community media exposure was 1.19 times more likely to have incomplete basic vaccination. This might be because as the level of media exposure decreases, women’s ability to access, share, understand health information, and seek healthcare services for their children will decrease. Similarly, low community-level maternal education was positively associated with incomplete basic vaccination of children. This indicates an expansion of immunization for children mainly related to maternal formal education and empowerment at the community level.

Moreover, the factor that has a potential association with incomplete basic vaccination is country-level income status. The finding from this study indicates children who are living in lower middle-income countries around the world have higher odds of having incomplete basic vaccination than those in low-income countries. This might be due to the increase in wealth status parallel with fast socio-economic growth in middle-income countries. This transition period resulted in preliminary independent healthcare service delivery with insufficient immunization coverage in lower middle-income countries (59), while low-income countries remained under non-governmental organizations with better immunization coverage and child health-seeking behavior.

## Strengths and limitations of the study

The strength of this study was the use of spatial regression analysis with a larger sample to detect significant hotspot areas of incomplete basic vaccination to plan location-specific and effective intervention programs. Another strength was the use of the DHS data by era before and after the Sustainable Development Goal endorsement, which gives recent information on incomplete basic vaccination. However, this study has some limitations. The study could not show cause–effect relationships due to the cross-sectional nature of DHS data. Second, SaTScan analysis detects only circular clusters. Therefore, irregularly shaped clusters may be missed. Third, the Kriging interpolation method is not mapped, even though it cannot show the interpolated values in non-stationary areas because of the lakes between continents. The GPS (latitude and longitude) data were taken from the enumeration area, which is displaced to 5 km in urban areas and 10 km in rural areas for privacy issues, and this might bias our spatial distribution.

## Conclusion

The spatial analysis showed that the spatial distributions of incomplete basic vaccination among children aged 12–23 months were significantly varied across the regions of the world. Resource-limited countries, such as Guinea, Ghana, Mauritania, Niger, Nigeria, Chad, Cameroon, Angola, Central Ethiopia, Madagascar, Haiti, Myanmar, Afghanistan, and Pakistan, were identified as significant hotspot areas where incomplete basic vaccination among children aged 12–23 months was high. This study found that age, marital status, maternal education, husband’s education, maternal occupation, media exposure, wealth index, ANC visits, birth order, place of delivery, mode of delivery, health insurance coverage, perception of distance from health facility, place of residence, community media exposure, community education, and country-level income status were significantly associated with children’s incomplete basic vaccination.

Therefore, it is recommended to increase healthcare, infrastructure accessibility, and maternal and child healthcare services across resource-limited countries globally with a high proportion of incomplete basic vaccinations.

The World Health Organization should emphasize public health initiatives aimed at supporting underprivileged families, including those with limited/no education and access to maternal healthcare services, which can reduce incomplete basic childhood vaccination rates and ultimately improve child survival.

Healthcare professionals should plan and implement different activities that increase timely vaccination initiation in collaboration with different media and expand immunization coverage to a large area.

Researchers entering this area should pay special attention to incomplete immunization among children living in hotspot areas of these countries.

Furthermore, a nationally representative primary study with a better study design is recommended to detect the cause–effect relationship of incomplete basic vaccination among children aged 12–23 months.

## Data Availability

The raw data supporting the conclusions of this article will be made available by the authors, without undue reservation.
